# Base excess and hematocrit predict response to indomethacin in very low birth weight infants with patent ductus arteriosus

**DOI:** 10.1186/s13052-019-0706-y

**Published:** 2019-08-22

**Authors:** Janardhan Mydam, Alok Rastogi, Zahra J. Naheed

**Affiliations:** 1 Division of Neonatology, Department of Pediatrics, John H. Stroger, Jr. Hospital of Cook County, 1969 W Ogden Avenue, Chicago, IL 60612 USA; 2 Division of Pediatric Cardiology, Department of Pediatrics, John H. Stroger, Jr. Hospital of Cook County, 1969 W Ogden Avenue, Chicago, 60612 IL USA

**Keywords:** Patent ductus arteriosus, Low birth weight, Acidosis, Preterm, Indomethacin, Hemodynamically significant PDA

## Abstract

**Background:**

The treatment of patent ductus arteriosus (PDA) in very low birth weight (VLBW) infants remains a challenge. The ability to predict which infants will respond to indomethacin could spare some from the risks of unnecessary medications. Our objective was to determine if indicators of acid-base homeostasis could predict response to indomethacin treatment for ductal closure, and thus help guide treatment decisions.

**Methods:**

We performed a retrospective analysis of medical records of VLBW (< 1500 g) neonates with hemodynamically significant PDA born at our institution between January 2009 and December 2012; all infants included in the study were treated with indomethacin for ductal closure within the first 2 weeks of life. We extracted data for a number of clinical variables including gestational age, birth weight, blood chemistries, surfactant use, hematocrit, and blood gas parameters. Our primary outcome measure was successful closure of PDA following the first round of indomethacin. Using variables that were significant on initial testing, we created multivariable regression models to determine the independent association of selected variables with indomethacin response.

**Results:**

Of the 91 infants included in the study, 62 (68%) responded to the first course of indomethacin with successful ductal closure. Multivariable regression modeling revealed that both base excess and hematocrit were independently associated with indomethacin response; odds of PDA closure increased with increasing base excess (OR [odds ratio]: 1.81; 95% confidence interval [CI]: 1.36–2.60) and increasing hematocrit (OR: 1.21; 95% CI: 1.01–1.45). The optimal cutoff value for base excess was − 4.56, with a sensitivity of 96.8% (95% CI: 89–100) and specificity of 79.3% (95% CI: 60–92); optimal cutoff value for hematocrit was 40, with 69.4% sensitivity (95% CI: 56–80) and 65.5% specificity (95% CI: 46–82).

**Conclusions:**

Base excess and hematocrit may be independent predictors of indomethacin response in VLBW infants with PDA. Low-cost and readily accessible, acid-base indicators such as base excess could help guide treatment decisions.

**Electronic supplementary material:**

The online version of this article (10.1186/s13052-019-0706-y) contains supplementary material, which is available to authorized users.

## Background

Management of hemodynamically significant patent ductus arteriosus (HSPDA) in VLBW (< 1500 g) infants remains a challenge. Increasingly, the decision to treat any given infant rests on a risk-benefit analysis, balancing the known risks of HSPDA with the known risks of treatment [1, 2]. Risks of untreated HSPDA in the VLBW infant are real; a substantial left-to-right shunt increases risk of bronchopulmonary dysplasia (BPD), necrotizing enterocolitis (NEC), renal dysfunction, and intraventricular hemorrhage [1].

Medical management (fluid restriction, diuretic therapy, and transfusion if indicated), pharmacologic therapy with cyclooxygenase (COX) inhibitors (indomethacin, ibuprofen) or acetaminophen, and surgical ligation are the current options for treating for HSPDA [3]. Both pharmacologic and surgical treatment are associated with adverse effects for the preterm infant. Side effects of indomethacin include increased risk of NEC [4], reduced renal function [5], and reduced cerebral blood flow [6]. Although surgical ligation achieves permanent ductal closure, ligation increases risk of BPD and retinopathy of prematurity [7], and is associated with other short-term and long-term morbidities [7].

The response to pharmacologic treatment varies, with the failure rate for indomethacin ranging from 13 to 40% [8–10]. Therefore, the ability to accurately predict indomethacin response would allow for selective medical treatment according to the likelihood of success, sparing some infants from exposure to unnecessary and potentially harmful courses of medication. A number of tools to predict or evaluate response to indomethacin therapy in VLBW infants have been explored, including brain natriuretic peptide (BNP) levels [11, 12], urinary N-terminal (NT)-proBNP [13], echocardiography [14, 15], plasma levels of indomethacin [16] and platelet count [9, 17, 18]. Although metabolic acidosis in preterm infants with PDA has been reported in the literature, with some suggesting that acidosis may be one of the earliest indicators of HSPDA [19], acid-base indicators have not been widely reported as predictive tools. To our knowledge, only 1 study has reported a marker of acid-base balance as a tool for predicting indomethacin response [20]. Therefore, we hypothesize that an indicator of acid-base balance, such as base excess, a low-cost, readily accessible biomarker, could potentially predict PDA response to indomethacin treatment.

The primary aim of our study was to identify bedside clinical indicators related to acid-base status that could accurately predict indomethacin-induced closure of HSPDA. As a secondary goal we explored the associations between a number of maternal, neonatal, and clinical variables and indomethacin response.

## Methods

### Study population

**Fig. 1:**
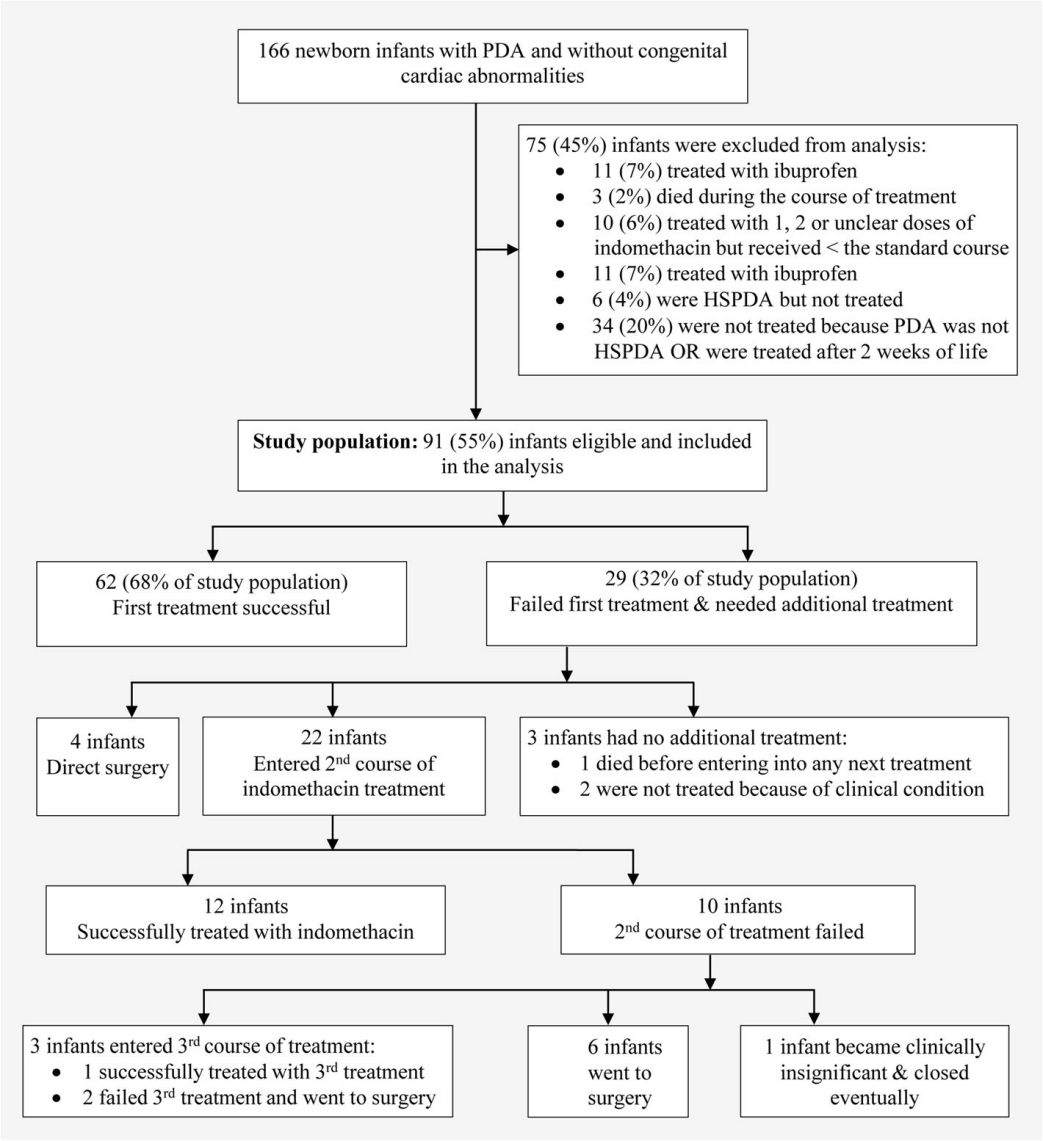
Flowchart indicating the patients considered for the retrospective analyses and their subsequent clinical course. HSPDA, hemodynamically significant patent ductus arteriosus

We conducted an observational, retrospective, case-controlled study of all VLBW (< 1500 g) infants born from January 2006 to December 2012 at John H Stroger Hospital of Cook County who had been diagnosed with PDA by echocardiogram during the first 2 weeks of life. We excluded infants whose PDA was not hemodynamically significant; infants who did not receive treatment; infants who were treated with ibuprofen; infants who were treated with indomethacin but received less than the standard course; infants with congenital cardiac abnormalities; and infants who died during the course of treatment (Fig. 1). An infant was considered to have hemodynamically significant PDA if they had a moderate-to-large PDA (DA diameter > 1.5 mm) [21] and left atrium (LA)/Aorta ratio ≥ 1.4 [22] and required ≥40% oxygen, where the oxygen requirement is attributable to PDA, based on radiologic evidence of increased blood flow to the lungs and not attributable to other respiratory conditions (e.g., respiratory distress syndrome, sepsis) [12, 14]. Our final analysis included only VLBW infants with HSPDA who had been treated exclusively with 3 doses of a first course of IV indomethacin within the first 2 weeks after birth, according to the following schedule:

1st dose = 0.2 mg/kg, and 2nd and 3rd doses = 0.1 mg/kg/dose if treatment initiated < 48 h after birth; 0.2 mg/kg/dose for 3 doses if initiated 2–7 days after birth; 1st dose = 0.2 mg/kg, and 2nd and 3rd doses = 0.25 mg/kg/dose if initiated 7–14 days after birth [23]. Doses were given every 12 to 24 h depending on infant’s urine output and biochemical renal function tests..

### Study variables

Our primary outcome was successful closure of PDA, determined by echocardiographic evidence of either 1) complete closure, or 2) DA < 0.5 mm and not seen in all views, with no evidence of increased pulmonary blood flow and normal LA/Ao (< 1.1). Infants who achieved PDA closure in response to indomethacin treatment were considered “responders,” and those whose PDA did not close were considered “non-responders.” Independent variables included demographic characteristics, antenatal steroids, use of vasopressors, birth weight, gestational age, surfactant use, Apgar score, admission temperature, blood gas parameters, hematocrit, serum sodium, blood urea nitrogen (BUN), creatinine, current weight, urine output, and daily total fluid intake. All the continuous variables we used in our analysis are averages of the 48- to 72-h time period prior to treatment of PDA. Blood gas measurements were obtained with Abbot’s iSTAT1 wireless analyzer.

### Statistical analyses

The categorical variables were described by total counts and percentages. Normally distributed continuous variables were reported by mean and standard deviation, while continuous variables with non-normal distribution were presented by median and range. We performed the χ^2^ test to compare categorical variables by outcome groups, the independent *t* test to compare normally distributed continuous variables by outcome groups, and the Wilcoxon-Mann-Whitney U-test to compare non-normally distributed continuous variables.

To identify associations between the independent variables and indomethacin response, we constructed a multivariable logistic regression model of the final pretreatment markers that were deemed significant by the χ^2^ test or the Mann-Whitney U test. For variables that showed a significant association or trended toward significance, we created comparative ROC curves to explore predictive capacity and then calculated sensitivity and specificity at various cut-off points. Finally, we determined optimal cut-off points. SAS 9.4 software was used for our analysis; *P* -value of < 0.05 was considered statistically significant. We performed power analysis before the initiation of this study. Power analysis indicated that a minimal total sample size of *n* = 64 was needed to demonstrate a mean difference of 2.5 in base excess (BE) between two groups with common variance of 3.5 to achieve a minimum power of 80% at level of significance 0.05.

## Results

We identified 166 VLBW infants who were diagnosed with PDA; 3 died, 11 were treated after 14 days, 10 were treated with either 1 or 2 dosages of indomethacin, 11 were treated with ibuprofen, and 40 infants were diagnosed with PDA but were not treated. Therefore, 75 infants were excluded from the study and 91 were eligible for analysis. All 91 infants included in the study had been diagnosed with HSPDA, were treated within 14 days after birth, and received 3 doses of indomethacin. Figure 1 (above) illustrates the selection process.

Table 1 describes the baseline characteristics of the 91 infants included in the study, classified by indomethacin response (responders and non-responders). Sixty-two infants (68%) responded to indomethacin with successful PDA closure while 29 (32%) did not respond. Only the mean birth weight, gestational age, and the 5-min Apgar were statistically different between the 2 groups. Table 2 shows the pretreatment laboratory biomarkers of responders and non-responders. There were significant differences in the pH (7.30 vs 7.27; *P* = 0.02), HCO_3_ levels (24.6 vs 20; *P*<0.001), base excess (− 0.8 vs − 5.7; *P<*0.001), hematocrit levels (42 vs 39; *P* = 0.001), weight (883 g vs 752 g; *P* = 0.04), and urine output (3.3 mL/kg/h vs 3.8 mL/kg/h; *P* = 0.04) between the 2 groups.

**Table 1:** Baseline characteristics of newborns with patent ductus arteriosus by treatment outcome

Variable^a^	Responders^b^ *N* = 62 (68%)	Non-responders *N* = 29 (32%)	*P* value
Maternal age (y) [mean (SD)]	25.7 (6.6)	24.4 (6.6)	0.35
Race / ethnicity [n (%)]			
White	5 (8.06)	2 (6.90)	1.00
Black	39 (62.90)	19 (65.52)	
Hispanic	18 (29.03)	8 (27.59)	
Sex [n (%)]			
Female	28 (45.16)	15 (51.72)	0.56
Gestational age (wk) [median (range)]	26.0 (22.0–30.0)	24.0 (21.0–28.0)	0.004*
GA (wk) [n (%)]			
< 28	48 (77.42)	26 (89.66)	0.16
PROM duration (h) [median (range)]	0 (0–360)	0 (0–168)	0.39
Antenatal steroid [n (%)]			
No	11 (17.74)	7 (24.14)	0.48
Chorioamnionitis^c^ [n (%)]			
Yes	7 (11.48)	7 (24.14)	0.13
Vasopressors use [n (%)]			
Yes	23 (37.10)	12 (41.38)	0.70
Multiple gestation [n (%)]			
Yes	9 (14.52)	4 (13.79)	1.00
No	53 (85.48)	25 (86.21)	
BW (g) [mean (SD)]	870 (246)	738 (232)	0.02^*^
Apgar at 5th min [n (%)]			
0–3	3 (4.84)	5 (17.86)	0.02^*^
4–6	14 (22.58)	11 (39.29)	
> 6	45 (72.58)	12 (42.86)	
Surfactant use [n (%)]			
Yes	60 (99.77)	29 (100.00)	1.00
Admission temperature (°C) [median (range)]	37 (34–38)	37 (33–38)	0.80
DOL at start of treatment (d) [median (range)]	4 (1–52)	5 (2–44)	0.40

*SD* standard deviation, *GA* gestational age, *PROM* premature rupture of membranes, *BW* birth weight, *DOL* day of life

^*^Significant (*P* < 0.05)

^a^For simplicity and readability, we have listed only one of the 2 possible options for binary variables

^b^Infants were considered responders if there was successful ductal closure following indomethacin treatment

^c^Variable had missing data

**Table 2:** Pretreatment bedside clinical parameters by treatment outcome

Variable	Responders *N* = 62 (68%) Mean (SD)	Non-responders *N* = 29 (32%) Mean (SD)	*P* value
Capillary blood gas			
pH	7.30 (0.04)	7.27 (0.05)	0.02
HCO_3_	24.6 (3.5)	20 (2.9)	< 0.001
Base excess	−0.8 (3.2)	−5.7 (3.3)	< 0.001
PCO_2_	49 (8)	48 (8)	0.86
Hematocrit	42 (4)	39 (5)	0.001
Na^+^	139 (5)	139 (4.3)	0.95
Fluid intake (mL/kg/d)	122 (16)	133 (22)	0.06
Weight (g)	883 (320)	752 (225)	0.04
UOP (m/kg/h)	3.3 (1)	3.8 (0.9)	0.04
BUN	35 (13)	42 (29)	0.94
Creatinine	0.9 (0.2)	0.9 (0.3)	0.52

*SD* standard deviation, *PCO*_*2*_ partial pressure of carbon dioxide, *PO*_*2*_ partial pressure of oxygen, *UOP* urine output, *BUN* blood urea nitrogen

Simple logistic regression analysis (Table 3) shows that gestational age (OR: 1.48; 95% CI: 1.13–1.93; *P* = 0.005) and Apgar score (0–3, 4–6, and > 6) at 5 min were significantly associated with indomethacin-induced closure. Compared to infants with 5-min Apgar score > 6, the odds of successful closure were 66% lower for infants with 5-min Apgar score 4–6 and 84% lower for infants with 5-min Apgar score of 0–3.

**Table 3:** Simple logistic regression analysis of successful closure of patent ductus arteriosus for baseline characteristics

Baseline characteristic	OR (95% CI)	*P* value
Birth weight	1.002 (1.00–1.01)	0.021
Gestational age	1.48 (1.13–1.93)	0.005
Apgar score at 5 min		
> 6	1.00	
0–3	0.16 (0.03–0.77)	0.02
4–6	0.34 (0.12–0.94)	0.04

*OR* odds ratio, *CI* confidence interval

Table 4 shows the results of the multivariable logistic regression analysis. We excluded pH and HCO_3_ in the regression model because of collinearity, since both of those variables are correlated with base excess. The logistic regression model showed that infants with higher base excess and higher hematocrit were more likely to respond to indomethacin treatment with ductal closure. After controlling for accompanying biomarkers, we did not find a significant association between either fluid intake or pretreatment weight and indomethacin. The odds of successful closure in response to indomethacin increased with increasing base excess (OR: 1.81; 95% CI: 1.36–2.60) and increasing hematocrit levels (OR: 1.21; 95% CI: 1.01–1.45).

**Table 4:** Multivariable logistic regression analysis of successful PDA closure following first course of indomethacin

Bedside clinical parameter^a^	OR (95% CI)	*P* value
Base excess	1.81 (1.36–2.60)	0.0001
Hematocrit	1.21 (1.01–1.45)	0.04
Fluid intake	0.97 (0.92–1.01)	0.16
Pretreatment weight	0.99 (0.99–1.00)	0.13

*OR* odds ratio, *CI* confidence interval

^a^pH and HCO_3_ were not included in the model because of collinearity with base excess

**Fig. 2:**
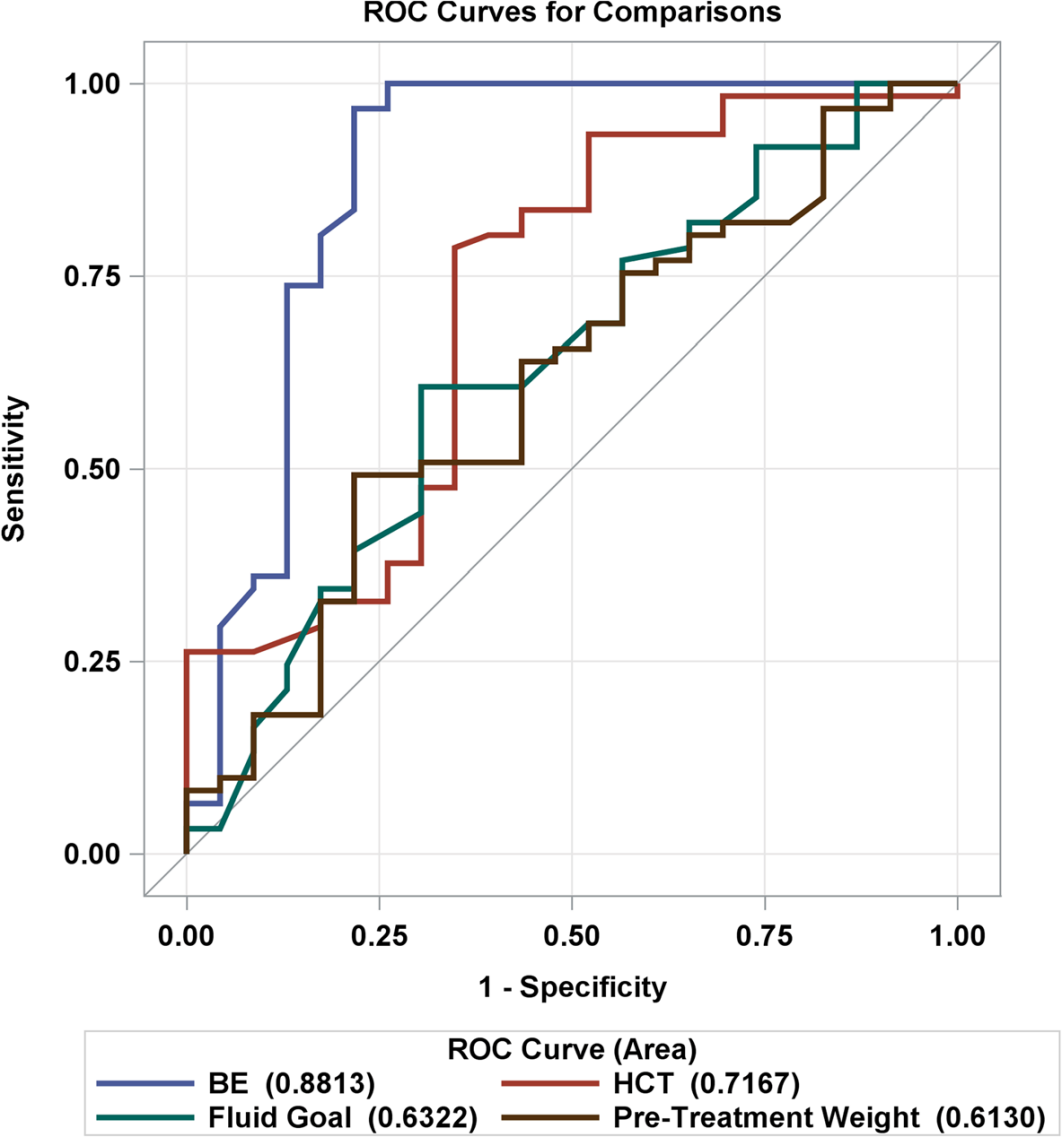
Receiver operating characteristic curve analysis for base excess, hematocrit, fluid goal and pretreatment weight

In order to quantify the predictive strength of the individual pretreatment laboratory biomarkers, we constructed comparative ROC curves for variables that were significant (base excess and hematocrit) or trended toward significance (fluid intake and pretreatment weight) on logistic regression (Fig. 2). According to the ROC curve analysis, the strength of hematocrit as an individual marker in predicting which infants would respond to indomethacin with successful PDA closure was fair (area under the curve [AUC] = 0.72), but higher than fluid intake (AUC = 0.63) and pretreatment weight (AUC = 0.61). Base excess proved to be the strongest predictor (AUC = 0.88) of indomethacin response.

We also calculated the sensitivities, specificities, predictive values, and likelihood ratios of base excess and hematocrit. The optimal cut-off value for base excess was − 4.56, providing 96.8% sensitivity, 79.3% specificity, a positive predictive value of 90.9%, and a negative predictive value of 90%, while the optimal cut-off value for hematocrit was 40, with a sensitivity of 69.35% and specificity of 65.5%, and positive and negative predictive values of 81.1 and 50%, respectively (Table 5; Additional file 1 shows values for all cutoffs for both base excess and hematocrit).

**Table 5:** Sensitivities, specificities, predictive values, and likelihood ratios for select base excess and hematocrit cutoff levels

Predicting variable	Cutoff level	Sensitivity (95% CI)	Specificity (95% CI)	Positive Predicting Value (95% CI)	Negative Predicting Value (95% CI)	Positive Likelihood Ratio (95% CI)	Negative Likelihood Ratio (95% CI)
Base excess	−6.56	100 (94,100)	44.83 (26,64)	79.49 (69,88)	100 (75,100)	1.81 (1.31,2.52)	0
	−4.56^a^	96.77 (89,100)	79.31 (60,92)	90.91 (81,97)	92 (74,99)	4.68 (2.29,9.55)	0.0407 (.01,.16)
	6.56	6.45 (2,16)	100 (88,100)	100 (40,100)	33.33 (24,44)	NA	0.0094 (.88,1.00)
Hematocrit	28	98.39 (91,100)	0 (0, 15)	67.78 (57,77)	0 (0,95)	0.98 (.95,1.02)	NA
	40^a^	69.35 (56,80)	65.52 (46,82)	81.13 (68,91)	50.00 (33,67)	2.01 (1.19,3.41)	0.47 (0.31,0.70)
	52	1.61 (0,.09)	96.55 (82,100)	50.00 (1,99)	31.46 (22,42)	0.47 (0.03,7.22)	1.02 (0.98,1.05)

*CI* confidence interval

^a^Optimal cutoff level; we have considered the value as the optimal cutoff for which the difference (sensitivity − specificity) is minimum

## Discussion

In this retrospective study we evaluated readily accessible, low-cost bedside clinical parameters as potential predictors of indomethacin response in VLBW infants with HSPDA. We are among the first to report that higher base excess and higher hematocrit level independently predict indomethacin success in this population. Approximately one-third (32%) of the infants in our study failed the first course of indomethacin, squarely within the range (13–40%) reported by similar studies [8–10, 12, 15, 17, 20, 24] and consistent with the estimated 25–30% overall failure rate of COX inhibitors [3]. Our failure rate underscores the value of predictive variables. Identifying which infants will respond to indomethacin treatment could spare a substantial number of infants unnecessary courses of medication.

Our finding that infants with acidosis, as indicated by lower base excess, were less likely to respond to indomethacin is consistent with Steiner et al., who found that lower arterial pH in the first 48 h of life predicted the need for surgical ligation following ibuprofen treatment [20]. Like our study, Steiner et al.’s predicting variable was independent of gestational age. Although gestational age is a known predictor of spontaneous closure, studies of its association with indomethacin-induced closure have been mixed [10, 17, 24–26].

We speculate that indomethacin might be less effective in an acidic environment because the acidosis favors multiple vasodilating forces. Both Moonen et al., in a study of chicken DA, and Celotto et al., in a study of rat thoracic aorta, demonstrated that extracellular acidosis induced vasodilation [27, 28], corroborating prior studies [29, 30]. Celotto et al. [28] found that the acidosis-induced vasodilation was not reversed by indomethacin, highlighting the role of forces other than prostaglandins in modulating vascular tone. Acidosis-induced vasodilation was reversed when nitric oxide (NO), calcium-activated potassium channels (K_Ca_), and ATP-sensitive K+ channels (K_ATP_) were blocked [28]. Moreover, acidosis increased NO production, and stripping the vessel of its endothelium revealed that endothelial NO and NO released from smooth muscle acted independently to relax vascular tone. They concluded that extracellular acidosis promotes vasodilation that is mediated by NO and/or potassium channels [28].

Evidence suggests that NO may be an important mediator in persistent ductal patency, particularly in preterm infants [31]. Seidner et al. [32] compared DA closure rates in newborn baboons who received only indomethacin with newborns who received indomethacin and an NO-blocking agent (*N*-nitro-L-arginine). The group who received both the NO blocker and indomethacin had 100% closure compared to 33% for the indomethacin-only group [32], supporting NO as a significant factor in maintaining ductal patency. We speculate that acidosis caused indomethacin resistance in our population because of its favorable effects on the vasodilating forces of NO [28]. We further speculate that VLBW infants with PDA who are acidotic may benefit from the addition of an NO-blocker to a COX inhibitor. Prospective studies to test this hypothesis are warranted.

Few studies have explored the relationship between hematocrit and PDA in neonates. Kalis et al. found that low hematocrit predicted indomethacin resistance, but suggested the finding could be a consequence of hemodilution due to the high volumes of IV fluids given to the infants [8]. It seems unlikely that our finding of an association between hematocrit and indomethacin response was related to dilution, because daily fluid intake did not differ significantly between responders and non-responders on regression analysis. More enlightening are studies of the relationship between hematocrit and spontaneous PDA closure. Kahvecioglu et al. found that levels of hemoglobin and hematocrit were significantly lower in their “open PDA” group vs their “closed PDA” group [33]. Similarly, Chen et al. concluded that lower hemoglobin levels after birth increased the risk of PDA in VLBW infants [34]. Because hematocrit directly influences oxygen levels, and high oxygen tension is important for ductal constriction [31], it is possible that infants in our study with higher hematocrit levels were more likely to respond to indomethacin treatment by PDA closure because of higher oxygen tension.

The independent association of base excess and hematocrit with indomethacin response in our study suggests these variables could be used to predict success or failure of pharmacologic treatment. Other predictive tools have been evaluated. BNP has been extensively studied as a biochemical variable associated with PDA closure [11–13, 35]. BNP, secreted by heart muscle in response to volume overload, is elevated in infants with PDA and levels are known to decrease upon PDA closure [35]. Attridge et al. used plasma BNP levels to gauge closure to guide treatment, and were able to reduce the number of indomethacin doses without increasing morbidity in their study population [11], and Hsu et al. found that high BNP levels predict poor indomethacin response [12]. These results are promising, though a recent systematic review reported that studies evaluating the diagnostic accuracy of BNP and NT-proBNP for HSPDA varied widely, with variances related to both assay characteristics and patient characteristics [36]. Weisz et al. concluded that the use of natriuretic peptides to manage PDA in preterm infants remains primarily a research tool pending resolution of these issues [37]. Czernik et al. demonstrated that levels of NT-proBNP in the urine predicted responsiveness to pharmacologic treatment [13]. Given that urine sampling is readily accessible and noninvasive, use of urinary NT-proBNP increases convenience over plasma/serum BNP measurements, though the tool is still limited by the need for assay kits and variability of results [36].

Echocardiography has also been explored as a predictive tool. Pees et al. successfully predicted the response to ibuprofen therapy in extremely preterm (< 28 weeks) infants by using serial echocardiograms to measure maximum flow velocity and PDA diameter [14]. Echocardiography is the gold standard for PDA diagnosis, but as a predictive tool it is limited by availability, expense, and the burden of multiple echocardiograms on the patients and providers. More promising, as far as broad application, are the results from Ahamad et al., who found that high platelet counts at time of treatment increased the likelihood of successful indomethacin therapy in infants with HSPDA by 50% [17] and Steiner et al., who found that low arterial pH levels in the first 48 h of life, predicted indomethacin resistance [20]. Like base excess and hematocrit, pH levels and platelet counts are low-cost and readily accessible variables.

Evaluating the strength of their models for predicting non-responsiveness to indomethacin, Czernik et al. found an AUC (area under the curve) of 83% for urinary NT-proBNP, and Hsu et al. found AUCs of 85 and 83% for BNP at baseline and at 24 h after first dose, respectively [12, 13]. Our models for predicting indomethacin success revealed an AUC of 87% for base excess, which compares favorably; we found that hematocrit was a weaker predictor (AUC of 71%). Regarding sensitivity and specificity of predicting variables, Steiner et al. reported that pH < 7.26 predicted subsequent surgical ligation following ibuprofen treatment with a sensitivity of 92% and specificity of 50% [20]. Hsu et al. established that a baseline BNP level > 1805 pg/mL predicted non-responsiveness to indomethacin requiring subsequent surgical ligation with a sensitivity of 88% and specificity of 87% [12]. Czernik et al. found a cut-off of > 210 μg/g urinary BT-ProBNP on day 14 had a sensitivity of 75% and specificity of 100% for predicting non-responsiveness to pharmacologic treatment with ibuprofen or indomethacin [13]. Again, our predictive variable base excess compares favorably; an optimal cut-off of > − 4.56 predicted successful PDA closure with a sensitivity of 96.8% and specificity of 79.3%. Hematocrit was less accurate; cut-off (> 40) yielded a sensitivity of 69% and specificity of 66% (Table 4). Our results suggest that beginning indomethacin therapy before base excess falls below the cut-off level (− 4.56) might improve the success rate of indomethacin-induced closure.

Although our discussion has focused on clinical parameters, other neonatal characteristics have been independently associated with response to indomethacin-induced PDA closure, including birth weight [9, 17], antenatal steroid exposure [10], age at time of treatment [24], male gender [17], race [10], intrauterine inflammation [26], respiratory distress syndrome [24, 26], and gestational age [10, 17, 25]. In our study, only birth weight, gestational age, and 5-min Apgar score were baseline neonatal characteristics that differed significantly between responders and non-responders on simple logistic regression analysis, but none of these variables remained significant after adjusting for other variables on multivariable logistic regression analysis.

Our study has some limitations. We restricted our final analysis to infants who were treated in the first 2 weeks after birth, which limits our ability to explore effects of timing of treatment on indomethacin response. Also, while our population of 91 infants with HSPDA is large compared to similar studies, ours remains a single-center, retrospective study. A larger, prospective study is warranted to confirm our findings and establish base excess as a valuable biochemical marker for the prediction of PDA closure in VLBW infants..

## Conclusions

In summary, our study shows that base excess could be used as a low-cost marker to accurately predict response to indomethacin in VLBW infants with HSPDA. We believe that considering base excess at the time of decision to initiate indomethacin therapy could improve the outcome of VLBW infants with HSPDA. We also hypothesize that metabolic acidosis itself might be enhancing vasodilation, perhaps via effects on NO; a prospective study of NO-blocking agents to promote closure in acidotic infants would be enlightening.

## Additional file

Additional file 1: **Table S1.** Sensitivities, specificities, positive and negative predicting values, and likelihood ratios for hematocrit cutoff levels. **Table S2.** Sensitivities, specificities, positive and negative predictive values, and likelihood ratios for base excess cutoff levels. These tables give complete data for all cutoff values for hematocrit (**Table S1.**) and base excess (**Table S2.**) as potential predictors for an infant’s response to indomethacin treatment for patent ductus arteriosus. (DOCX 19 kb)

## Data Availability

The datasets used and/or analyzed during the current study are available from the corresponding author upon reasonable request.

## Abbreviations

PDA: Patent ductus arteriosus
VLBW: Very low birthweight
HSPDA: Hemodynamically significant PDA
NEC: Necrotizing enterocolitis
COX: Cyclooxygenase inhibitor
BNP: Brain natriuretic peptide
BUN: Blood urea nitrogen
ROC: Receiver operating characteristic
AUC: Area under the curve
NO: Nitric oxide
NT-proBNP: N-terminal-proBNP
K_Ca_: Calcium-activated potassium channels
K_ATP_: ATP-sensitive K+ channels
BPD: Bronchopulmonary dysplasia
