# Supplementary material for: Base excess and hematocrit predict response to indomethacin in very low birth weight infants with patent ductus arteriosus
Source: Ital J Pediatr. 2019 Aug 22;45:107. doi: 10.1186/s13052-019-0706-y (PMC6704716; doi:10.1186/s13052-019-0706-y)
Supplement: Supplementary file 1 — Table S1. Sensitivities, specificities, positive and negative predicting values, and likelihood ratios for hematocrit cutoff levels. Table S2. Sensitivities, specificities, positive and negative predictive values, and likelihood ratios for base excess cutoff levels. These tables give complete data for all cutoff values for hematocrit (Table S1.) and base excess (Table S2.) as potential predictors for an infant’s response to indomethacin treatment for patent ductus arteriosus. (DOCX 19 kb) [file 13052_2019_706_MOESM1_ESM.docx]

**Table S1.** Sensitivities, specificities, positive and negative predicting values, and likelihood ratios for hematocrit cutoff levels

| **HCT cutoff level** | **Sensitivity  (95% CI)** | **Specificity  (95% CI)** | **Positive**  **Predicting**  **Value**  **(95% CI)** | **Negative**  **Predicting**  **Value**  **(95% CI)** | **Positive**  **Likelihood**  **Ratio**  **(95% CI)** | **Negative**  **Likelihood**  **Ratio  (95% CI)** |
| --- | --- | --- | --- | --- | --- | --- |
| 28 | 98.39  (91,100) | 0  (0, 15) | 67.78  (57,77) | 0  (0,95) | 0.98  (.95,1.02) | NA |
| 31 | 96.77  (89,100) | 79.31  (60,92) | 67.42  (57,77) | 0  (0,80) | 0.97  (.92,1.01) | NA |
| 34 | 96.77  (89,100) | 17.24  (6,36) | 71.43  (61,81) | 71.43  (29,96) | 1.17  (0.98,1.39) | 0.19  (0.03,1.01) |
| 37 | 90.32  (80,96) | 48.28  (29,67) | 78.87  (68,88) | 70.00  (46,88) | 1.74  (1.22,2.51) | 0.20  (0.09,0.46) |
| 40 | 69.35  (56,80) | 65.52  (46,82) | 81.13  (68,91) | 50.00  (33,67) | 2.01  (1.19,3.41) | 0.47  (0.31,0.70) |
| 43 | 33.87  (22,47) | 75.86  (56,90) | 75.00  (55,89) | 34.92  (23,48) | 1.40  (.67,2.92) | 0.87  (0.71,1.06) |
| 46 | 12.90  (06,24) | 96.55  (82,100) | 88.89  (52,100) | 34.15  (24,45) | 3.74  (0.49,28.54) | 0.90  (0.82,1.00) |
| 49 | 6.45  (2, 16) | 96.55  (82,100) | 80.00  (28,100) | 32.56  (23,44) | 1.87  (0.22,16.00) | 0.97  (.91,1.04) |
| 52 | 1.61  (0,.09) | 96.55  (82,100) | 50.00  (1,99) | 31.46  (22,42) | 0.47  (0.03,7.22) | 1.02  (0.98,1.05) |

CI, confidence interval; HCT, hematocrit

**Table S2.** Sensitivities, specificities, positive and negative predictive values, and likelihood ratios for base excess cutoff levels

| **BE cutoff levels** | **Sensitivity  (95% CI)** | **Specificity  (95% CI)** | **Positive**  **predicting**  **value**  **(95% CI)** | **Negative**  **predicting**  **value**  **(95% CI)** | **Positive**  **likelihood**  **ratio**  **(95% CI)** | **Negative**  **likelihood**  **ratio**  **(95% CI)** |
| --- | --- | --- | --- | --- | --- | --- |
| −6.56 | 100  (94,100.00) | 44.83  (26,64) | 79.49  (69,88) | 100  (75,100) | 1.81  (1.31,2.52) | 0 |
| −4.56 | 96.77  (89,100) | 79.31  (60,92) | 90.91  (81,97) | 92  (74,99) | 4.68  (2.29,9.55) | 0.0407  (.01,.16) |
| −2.56 | 62.90  (50,75) | 86.21  (68,96) | 90.70  (78,97) | 52.08  (37,67) | 4.56  (1.80,11.56) | 0.4303  (.31,.60) |
| 0.56 | 32.26  (21,45) | 96.55  (82,100) | 95.24  (76,100) | 40.00  (28,52) | 9.35  (1.32,66.37) | 0.7016  (.59,.84) |
| 2.56 | 17.74  (09,30) | 96.55  (82,100) | 91.67  (62,100) | 35.44  (25,47) | 5.14  (.70,37.98) | 0.852  (.76,.96) |
| 4.56 | 09.68  (04,20) | 96.55  (82,100) | 85.71  (42,100) | 33.33  (23,44) | 2.81  (.35,22.25) | 0.9354  (.86,1.02) |
| 6.56 | 06.45  (02,16) | 100  (88,100) | 100  (40,100) | 33.33  (24,44) | NA | 0.0094  (.88,1.00) |

BE, base excess; CI, confidence interval
